# 2H-NbS_2_ film as a novel counter electrode for meso-structured perovskite solar cells

**DOI:** 10.1038/s41598-018-25449-x

**Published:** 2018-05-04

**Authors:** Feng Shao, Zhangliu Tian, Peng Qin, Kejun Bu, Wei Zhao, Li Xu, Deliang Wang, Fuqiang Huang

**Affiliations:** 1 Hefei National Laboratory for Physical Sciences at the Microscale, University of Science and Technology of China, Hefei, 230026 China; 2 State Key Laboratory of High Performance Ceramics and Superfine Microstructure, Shanghai Institute of Ceramics, Chinese Academy of Sciences, Shanghai, 200050 China; 3 State Key Laboratory of Rare Earth Materials Chemistry and Applications, College of Chemistry and Molecular Engineering, Peking University, Beijing, 100871 China; 4 Material Laboratory of State Grid Corporation of China, State Key laboratory of Advanced Transmission Technology, Global Energy Interconnection Research Institute, Beijing, China

## Abstract

We report the use of 2H-NbS_2_ film as a novel counter electrode in perovskite solar cells fabricated with a cold isostatic pressing method. The 2H-NbS_2_ film, which was prepared through an exfoliation method followed by restacking from Li_x_NbS_2_ powder, shows high electrical conductivity of 8.7 × 10^3^ S cm^−1^ and work function of 5.20 eV. The two-dimensional transition metal dichalcogenide was used for the first time as a counter electrode in meso-structured perovskite solar cells. Through this process, we demonstrated a new alternative to noble metals. The perovskite solar cell base on the 2H-NbS_2_ counter electrode showed an open-circuit voltage of 1.046 V, comparable to that of gold, and a power conversion efficiency of 8.3%.

## Introduction

Perovskite solar cells (PSCs) have been intensively investigated as a promising candidate for the low cost photovoltaic technology since the first report by Miyasaka *et al*. in 2009^1^. Tremendous efforts have been made in the investigation of crystal growth and intrinsic properties of the perovskite materials^2–6^ as well as the improvement of device performance and stability^7–9^. The latest certified power conversion efficiencies (PCEs) of the small-area perovskite solar cells (0.0946 cm^2^) and mini-modules (36.1 cm^2^) have been reported to be 22.1%^10^ and 12.1%^11^, respectively, approaching those of the commercial polycrystalline silicon, CdTe, and CuIn_(1−x)_Ga_x_Se_2_ solar cells^12^. In spite of the rapid advances of the perovskite solar cells, there are concerns with respect to the device degradation and large-scale commercialization^13^.

The reactivity of the perovskite material with the back contact has been suggested as an important factor to influence the properties of devices^14–16^. Gold (Au) has been widely used as the counter electrode in PSCs due to its less chemical reactivity, great conductivity and reflectivity, as well as high work function. However, chemical reactions are still observed at the interface^17^. This, together with its high price and the requirement for energy-consuming vacuum processing technique, restrict the further application of noble metals to some extent. To lower the fabrication cost of the counter electrode, it is essential to develop novel alternatives for the replacement of noble metals. The carbon-based materials are promising candidates due to the better stability and low cost^18^. The electrodes with different morphology and composition, such as carbon black^19^, graphene^20,21^, graphene oxide^22^, carbon nanotubes^23,24^, carbon paint^25^, and carbon cloth^26^, have been intensively investigated and the PCEs up to 16% have been achieved^27^. The application of carbon-based electrode in the PSCs provides a new direction for material selection. Up to now, the counter electrode materials used in PSCs are limited to metals and the carbon family. New materials for counter electrode need to be discovered.

The layered transition metal dichalcogenides (TMDs) with the general formula of MX_2_, where M is a transition metal and X is a chalcogen atom (S, Se or Te), have attracted much attention in recent years due to their excellent electronic properties, great mechanical flexibility, and partial optical transparency. Benefitting from the well-defined crystalline structure with few surface dangling bonds, the TMDs exhibit variable electronic properties such as superconducting (e.g., NbS_2_), magnetic (e.g., CrSe_2_), insulating (e.g., BN), topological insulating (e.g., Bi_2_Te_3_), and thermoelectric (e.g., Bi_2_Te_3_) phenomena^28^. The weakly bonded atomic layers in these materials facilitate the exfoliation from bulk to thin layers, thus providing a potential application in many fields such as lithium ion battery^29^. In this work, we used a cost-effective and flexibly metallic 2H-NbS_2_ film as a counter electrode in perovskite solar cells. The cold isostatic pressing (CIP) method was successfully developed instead of the conventional thermal evaporation or solution based printing techniques^30^. The work function of the prepared NbS_2_ film was measured to be 5.20 eV, matching the highest occupied molecular orbital energy level of the used hole transporting material 2,2′,7,7′-tetrakis(*N*,*N*-di-*p*-methoxyphenylamine)-9,9′-spirobifluorene (spiro-OMeTAD, 5.22 eV)^31,32^. This, together with a high electrical conductivity (8.7 × 10^3^ S cm^−1^), indicated efficient charge extraction and collection at the counter electrode. The PSC with the configuration of FTO/TiO_2_/MAPbI_3_/spiro-OMeTAD/NbS_2_ has a maximum power conversion efficiency of 8.3% from our measurements. We believe that with further optimization of the material and the fabrication technique, the performance of our devices can be further improved.

## Results and Discussion

**Figure 1:**
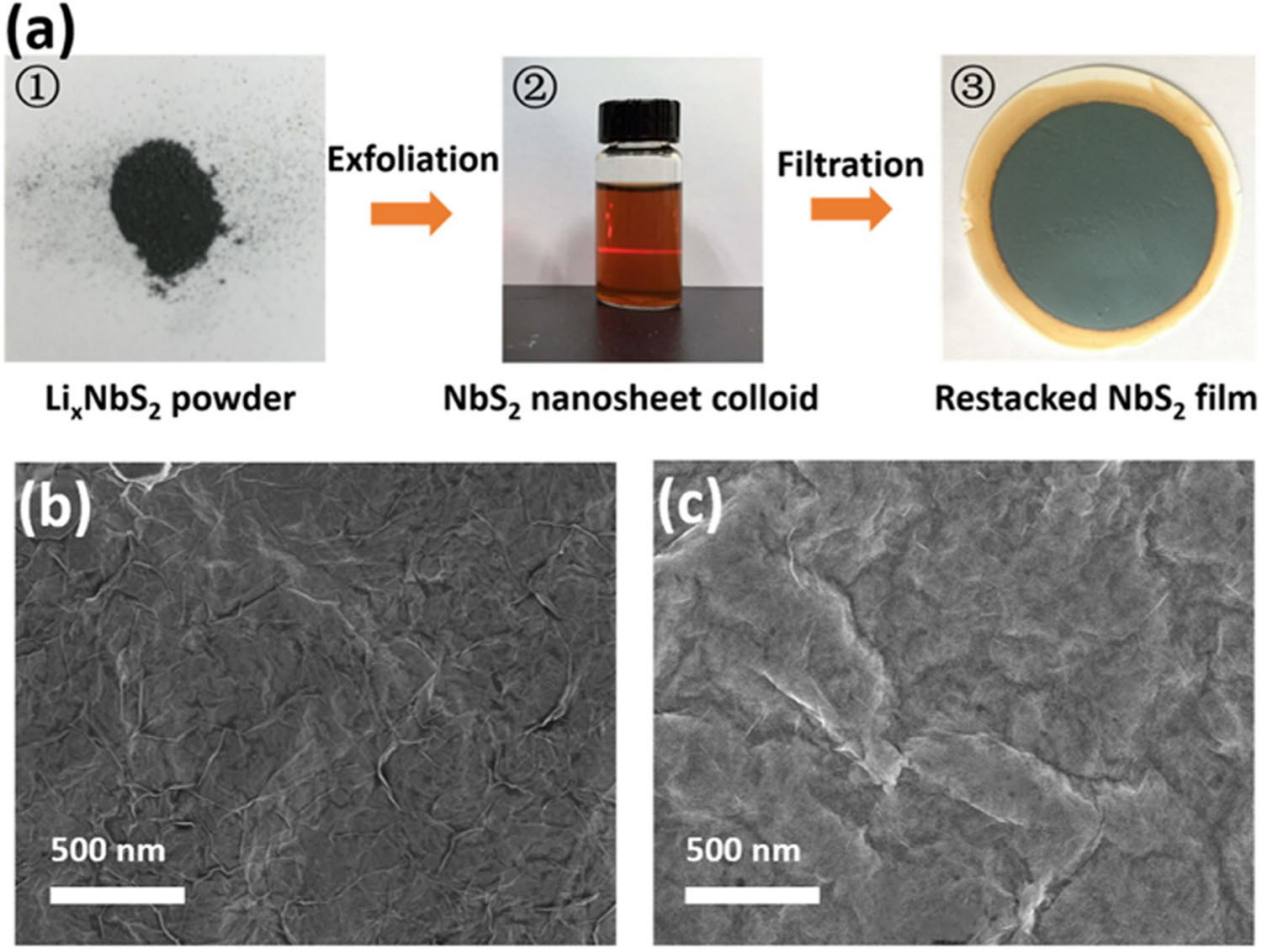
(**a**) The fabrication of NbS_2_ film from the Li_x_NbS_2_ power. Top-view FESEM images of the NbS_2_ film before (**b**) and after (**c**) the cold isostatic pressing treatment.

Figure 1a shows the multi-step process for the preparation of NbS_2_ thin film^33^. NbS_2_ powder was firstly synthesized through the conventional solid-state reaction (SSR) from Nb and S^34^. The powder was then reheated with Li_2_S and Nb for the intercalation of Li cation within the NbS_2_ layers. Exfoliation of the Li_x_NbS_2_ in hydrochloric acid aqueous solution led to the exchange of Li^+^ with H^+^, forming a homogeneous NbS_2_ nanosheet colloid. From the atomic force microscopic (AFM) and high-resolution TEM (HRTEM) images, the prepared NbS_2_ nanosheets were found to be just one to two layers in most cases with the thickness of around 1.1 nm in the hexagonal lattice structure (Fig. S1a and b). A lattice spacing of 2.90 Å is assigned to the 2H-NbS_2_ (100) plane. The final NbS_2_ thin film was fabricated by restacking the prepared nanosheets through vacuum filtration, and the film thickness can be easily tuned by controlling the amount of the colloidal solution. The prepared NbS_2_ film exhibits many wrinkles as shown in the scanning electron microscopy (SEM) image in Fig. 1b.

**Figure 2:**
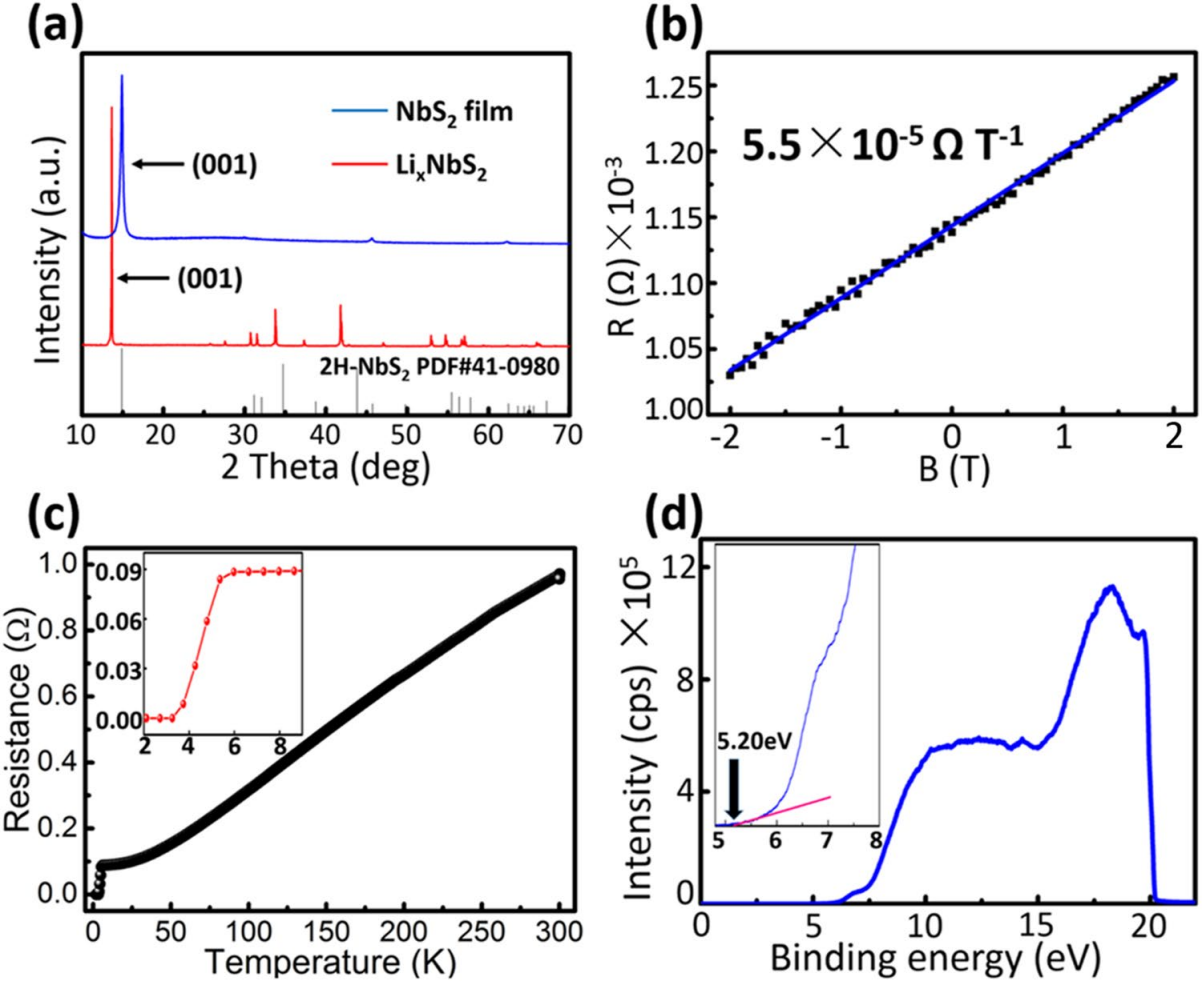
(**a**) XRD patterns of Li_x_NbS_2_ powder and the NbS_2_ film, (**b**) hall resistance (R) as a function of magnetic field (B) at 300 K, (**c**) temperature-dependent resistance curve and (**d**) the UPS spectrum of the prepared NbS_2_ film. The thickness of NbS_2_ film is 600 nm. The inset in (**c**,**d**) are the magnifications of local details.

The X-ray diffraction (XRD) pattern of the final NbS_2_ film was measured to check the influence of the Li exfoliation. As shown in Fig. 2a, the (001) plane is shifted from 13.7° to 14.9° due to the decreased inter-planar spacing after extracting the Li^+^. The only strongest peak at 14.9° confirms the 2 H phase and the good lattice orientation after restacking. Figure 2b shows the Hall-effect curve of the prepared NbS_2_ film with a thickness of 600 nm. By fitting with a linear function with a slope of 5.5 × 10^−5^ Ω T^−1^, a carrier concentration of 1.9 × 10^21^ cm^−3^ is extracted. To further investigate the charge transportation properties, a temperature-dependent resistance variation was measured using a four probe method (Fig. 2c). The prepared NbS_2_ film exhibits a typical metallic feature with a gradual drop of resistance with the decrease of temperature. A clear superconducting transition is observed when the temperature moves down to 6 K, consistent with previous reports^35,36^. The conductivity of the NbS_2_ film at 300 K is calculated to be 8.7 × 10^3^ S cm^−1^, which is lower than that of gold (4.6 × 10^5^ S cm^−1^). However, it is about 15 times higher than that of the assembled graphene (550 S cm^−1^)^37^ and 290 times higher than that of the carbon cloth based electrode (30 S cm^−1^)^26^, indicating that our NbS_2_ films prepared by the SSR method is a good candidate as a counter electrode in PSCs. The DFT calculation was performed to study the electronic structures of 2H-NbS_2_. No band gap in the bulk 2H-NbS_2_ was observed, which further confirmed its metallic character (Fig. S2). A work function of 5.20 eV was obtained from the UPS spectrum, slightly higher than that of Au (Figs 2d and S4). Close work functions between NbS_2_ and the spiro-OMeTAD hole transporting material (HTM) indicate that the NbS_2_ electrode can provide efficient charge separation at the interface.

**Figure 3:**
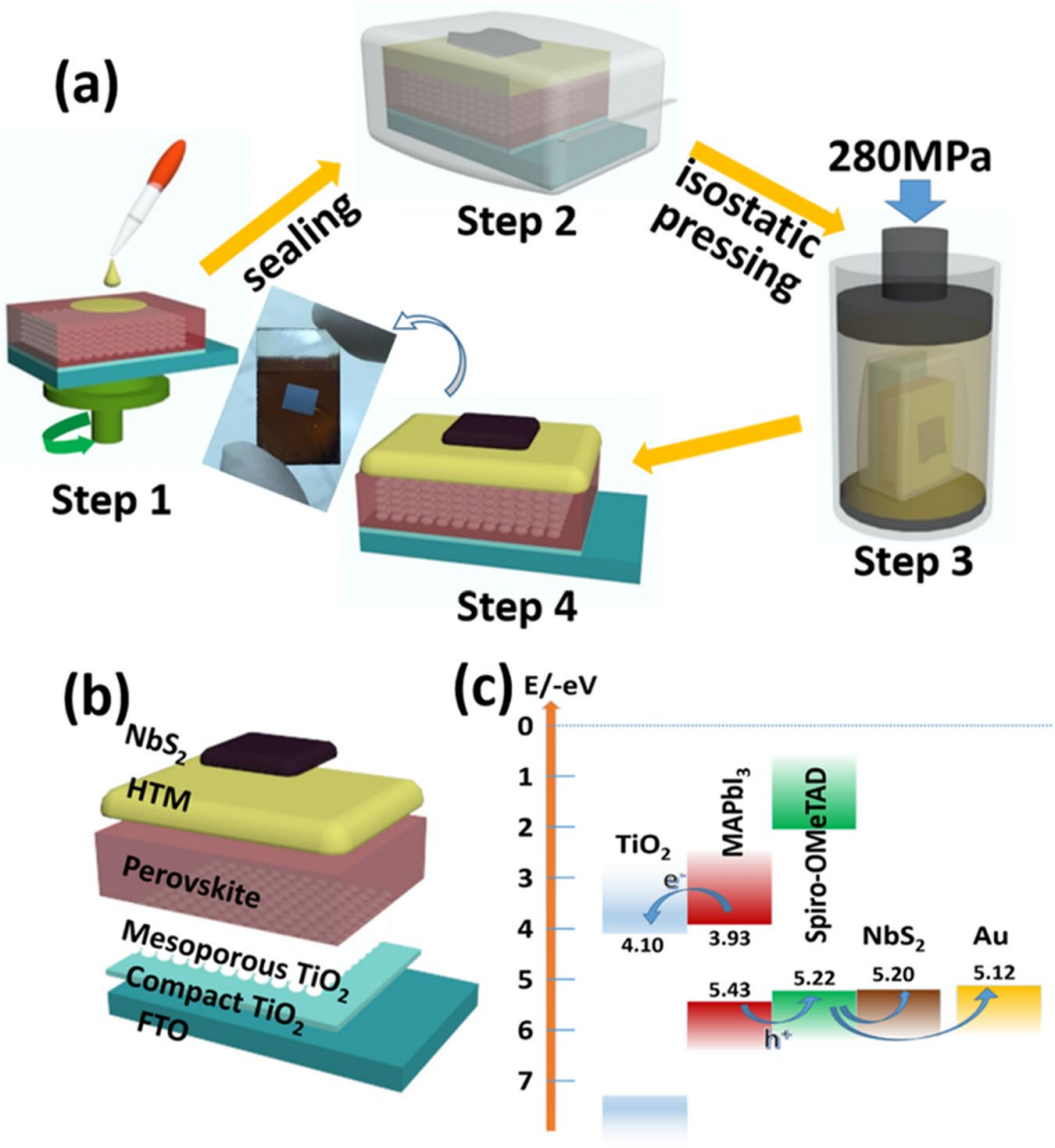
(**a**) Scheme of the device fabrication process, (**b**) the device architecture and (**c**) energy level diagram of FTO/TiO_2_/MAPbI_3_/spiro-OMeTAD/NbS_2_ solar cells.

The exfoliation fabrication process of the device is illustrated in Fig. 3a. The FTO/TiO_2_/MAPbI_3_ electrode was fabricated according to earlier publications^26^. The spiro-OMeTAD solution was then spin-coated on the top of the fresh prepared perovskite layer as a HTM. In the counter electrode part, the prepared NbS_2_ film was transferred to the HTM surface, followed by sealing the whole device into a polyethylene envelope under vacuum. The envelope was put into the CIP chamber full of hydraulic oil, pressed with a pressure of 280 MPa at room temperature for a couple of minutes. After this high pressure treatment, the NbS_2_ film was tightly plastered onto the spiro-OMeTAD surface (Figs 1c and S3a). No breakages or ruptures were observed under the mechanical pressure treatment from the SEM images. The NbS_2_ film was even smoother and more compact comparing with the fresh one (Fig. 1c). Therefore, as a less energy consuming and easy preparation technique, the CIP could be a new method to be used on flexible films for device preparation. The whole device architecture and the energy level diagram are shown in Fig. 3b,c^31,32^. The TiO_2_ and spiro-OMeTAD layers work as the electron and hole collector, respectively, with appropriate energy levels for efficient charge extraction.

The influence of NbS_2_ thickness on the device performance was investigated. The NbS_2_ films with the thickness of 300 nm, 600 nm and 1200 nm were prepared. The best photovoltaic performance was obtained while using the 600 nm film (Fig. S5 and Table S1). It was found that all the performance parameters initially increase with the increase of the NbS_2_ film thickness, followed by a drop at film thickness more than 600 nm. Considering the balance between resistance and conductivity, the 600 nm NbS_2_ film is chosen in our further studies. For the CIP technique, the pressure is an important factor because the perovskite layer can crack during this process. To check this effect, traditional mesoporous based PSCs with or without spiro-OMeTAD were fabricated and the photovoltaic properties were tested before and after the CIP (280 MPa) treatment. As shown in Fig. S6a and b, no obvious changes were observed after the CIP treatment in both cases. It proves that 280 MPa CIP treatment does not have much effects on the perovskite/HTM layer. A lower pressure of 100 MPa was tested under the same conditions for comparison. As shown from the statistics data, the higher pressure (280 MPa) is slightly better (Fig. S6d). The sequential operation starting from 100 MPa, followed by 280 MPa for one more time did not make any difference (Fig. S6c).

**Figure 4:**
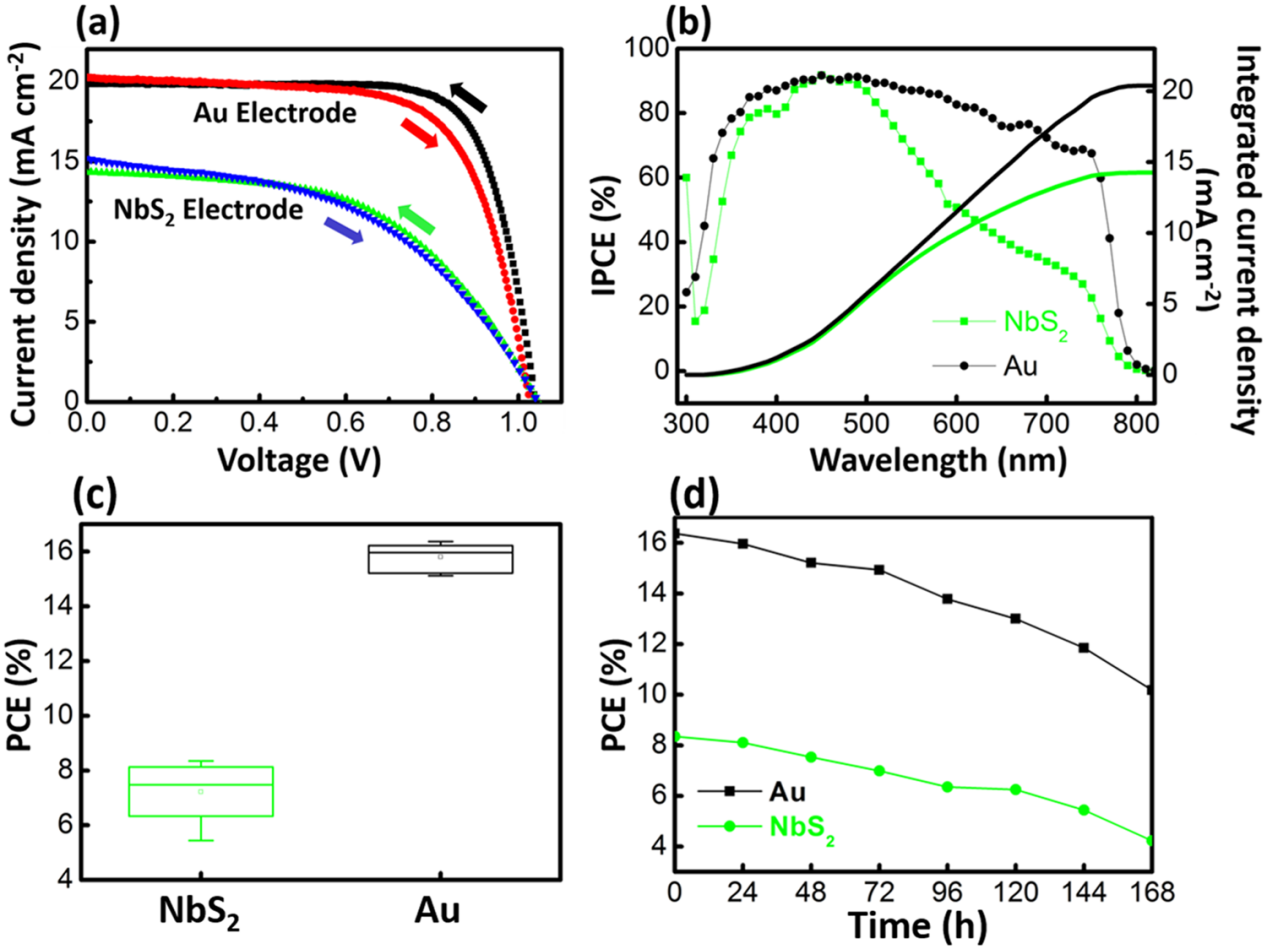
(**a**) Current-voltage (*J*-*V*) curves of the PSCs using NbS_2_ and Au as the counter electrode, measured by the forward and reverse scans under AM 1.5 full sun illumination. (**b**) The IPCE spectra of the corresponding devices. The integrated photocurrents calculated are 14.26 and 20.39 mA cm^−2^ for NbS_2_ and Au, respectively. (**c**) Statistics of PCE. Each box represents the distribution of 10 devices prepared under the same conditions. (**d**) Degradation of PCE of the unencapsulated two best-performed devices under the ambient condition at room temperature (24 °C).

Figure 4a demonstrates the current-voltage characteristics of one of the best performing devices based on the NbS_2_ and Au counter electrodes at a scan rate of 10 mV s^−1^. The Au electrode was used as a reference for comparison. The photovoltaic parameters are summarized in Table 1. The reference device based on the Au counter electrode shows a short-circuit photocurrent density (J_sc_) of 19.85 mA cm^−2^, an open-circuit voltage (V_oc_) of 1036 mV, and a fill factor (FF) of 0.76, resulting in a PCE of 16.37% under standard AM 1.5 G illumination. Under the same condition, the NbS_2_ based device displayed slightly higher V_oc_ of 1046 mV, but lower J_sc_ (14.34 mA cm^−2^) and FF (0.53), which gave a PCE of 8.31%. The lower J_sc_ and FF are mainly due to the large series resistance related to the NbS_2_ counter electrode (302.8 Ω) comparing with that of Au (29.4 Ω) either from the NbS_2_/HTM interface or the NbS_2_ itself, which then lead to an ineffective charge collection. A small hysteresis is observed in the J-V curves between the forward and reverse scans for NbS_2_. A slightly low FF leads to a 0.42% drop of PCE in the forward scan. From the incident photon-to-current conversion efficiency (IPCE) spectra, the integrated short-circuit current densities are in agreement with those obtained from the J-V curves (Fig. 4b). The device statistics based on two different counter electrodes over several batches are shown in Figs 4c and S7a. The average PCEs for PSCs based on NbS_2_ and Au electrodes are 7.22 ± 1.02%, and 15.80 ± 0.45%, respectively (shown in the parenthesis in Table 1). To evaluate the stability of the devices, two unsealed best-performed solar cells based on NbS_2_ and Au counter electrode were kept in ambient atmosphere (humidity > 50%) at room temperature (24 °C). The J-V curves were measured every 24 h (Figs 4d and S7b). The performance of both cells drops gradually with time, showing 50–60% of their initial value after a period of 7 days.

**Table 1:** The photovoltaic parameter of the perovskite solar cells based on NbS_2_ and Au counter electrode under AM 1.5 G illumination.

Sample	Scan Direction	*V*_oc_ (mV)	*J*_sc_ (mA cm^−2^)	*FF*	*η* (%)
Au Electrode	Reverse	1036 (1.039 ± 0.006)	19.85 (19.89 ± 0.37)	0.76 (0.73 ± 0.02)	16.37 (15.80 ± 0.45)
	Froward	1025	20.26	0.68	14.76
NbS_2_ Electrode	Reverse	1046 (1.020 ± 0.031)	14.34 (14.11 ± 1.57)	0.53 (0.48 ± 0.06)	8.31 (7.22 ± 1.02)
	Forward	1043	15.13	0.48	7.89

The average values shown in parentheses were obtained from 10 devices respectively. The active area of the devices is 0.16 cm^2^.

In summary, we took advantage of a general solid-state strategy to prepare the single-layered 2H-NbS_2_ nanosheets. Through simple filtration of the nanosheet colloid, the flexible NbS_2_ film with a high electrical conductivity of 8.7 × 10^3^ S cm^−1^ and a work function of 5.20 eV was obtained. The cold isostatic pressing method was firstly used to combine the prepared NbS_2_ film over the organic hole transporting layer for the preparation of perovskite solar cells. Based on this new counter electrode, the device showed a power conversion efficiency of 8.3% in our tests, and a comparable V_oc_ (1046 mV) comparing with that of gold. The two-dimensional transition metal dichalcogenide film is used as a counter electrode in perovskite solar cells, avoiding the conventional use of expensive noble metals and the high-energy consuming thermal evaporation process. Although both the TMD materials and the isostatic pressing technique need to be further optimized to improve the performance, this method provides an alternative way for the selection of new type of electrodes for the photovoltaic devices.

## Methods

### NbS_2_ film preparation

The NbS_2_ powder was synthesized by heating the stoichiometric ratio of niobium (99.8%, Alfa Aesar) and sulfur (99.999%, Alfa Aesar) in an evacuated quartz tube at 900 °C for 20 h^33^. The prepared NbS_2_, together with Li_2_S and Nb in a molar ratio of 3:2:1, were reheated through solid state reaction at 800 °C for 10 h, for the preparation of Li_x_NbS_2._ The NbS_2_ nanosheets were obtained by chemical exfoliation of Li_x_NbS_2_ in the hydrochloric acid (HCl) aqueous solution. In a typical exfoliation process, 30 mL of 1 M HCl was added to 200 mL of water. Then, 5 mg of Li_x_NbS_2_ was added. After ultrasonication and stirring for 15 min, the homogeneous NbS_2_ nanosheet colloid was obtained without any sediments inside, which was dialyzed several times to remove the impurities. The final NbS_2_ film was obtained through vacuum filtration of the prepared NbS_2_ nanosheets colloid using a PES (Sterlitech, Kent, WA) filter paper. In a typical experiment, 200 mL NbS_2_ solution with the concentration same as above led to a membrane thickness of about 300 nm.

### Fabrication of the Perovskite Solar Cells

The chemically etched (zinc powder and 4 M HCl) FTO conducting glass was sequentially cleaned by 2% Hellmanex water solution, deionized water, ethanol, and acetone. A 30–50 nm compact TiO_2_ layer was then deposited by spray pyrolysis at 450 °C from a precursor solution of titanium diisopropoxidebis(acetylacetonate) in anhydrous ethanol, using oxygen as the carrier gas. The mesoporousTiO_2_ layer was prepared by spin coating the TiO_2_ paste (Dyesol 30NRD, dilute 7 times in EtOH) at 4000 rpm for 20 s, followed by sintering at 500 °C for 30 min in air. The mixture of methylammonium iodide (MAI, Dyesol) and PbI_2_ (TCI) was dissolved in dimethyl sulfoxide (DMSO) with a molar ratio of 1:1.05. After stirring at 70 °C for 1 h, the precursor solution was spin-coated in a two-step procedure at 1000 and 7000 rpm for 10 and 40 s, respectively. During the second step, 200 μL of 3α-Trifluorotoluene was poured on the spinning substrate 15 s before the end of the procedure. The substrates were then annealed at 100 °C for 1 h in a nitrogen filled glove box. The HTM solution, consisting of 0.06 M Spiro-OMeTAD, 0.03 M bis(trifluoromethylsulphonyl)-imide lithium salt (Li-TFSI, Sigma-Aldrich), 0.006 M tris(2-(1H-pyrazol-1-yl)−4-tert-butylpyridine)–cobalt(III)tris(bis(trifluoromethyl-sulphonyl)imide) (FK209, Dyesol) and 0.2 M 4-tert-butylpyridine (TBP, Sigma-Aldrich) in chlorobenzene, was spin-coated on the top of perovskite layer at 2500 rpm for 15 s. Finally, the NbS_2_ film was cut off and transferred on the top of HTM with the help of a cling film. The whole device was sealed in a polyethylene envelope under a vacuum of 0.1 Torr, followed by putting into the hydraulic oil of the isostatic pressing machine for a high-pressure treatment. For comparison, 100 nm Au was deposited on the top of the HTM layer by thermal evaporation as a back contact as the reference.

### Measurements and Characterization

The X-ray diffraction patterns were measured by the Bruker D8 Focus instrument with a monochromatized source of Cu Kα1 radiation (λ = 0.15405 nm) at 1.6 kW (40 kV, 40 mA). The top-view field emission scanning electron microscopy (FESEM) and transmission electron microscopy (TEM) images were obtained using ZEISS SUPRA 55 microscope and the JEOL 2011microscope (2000 kV), respectively. Topographic imaging of the NbS2 nanosheet was performed using a Bruker Dimension Icon AFM equipped with a NSC14/Cr-Au with a spring force constant of 1−5 N/m and tip radius < 35 nm. The AFM was operated in the ‘tapping’ mode. The working point was 20746 nm, scan rate was 1.0 Hz. The temperature-resistance curve and Hall-effect curve of the NbS_2_ film with a thickness of 600 nm were characterized by the physical property measurement system (PPMS) of Quantum Design. Temperature variation of the resistance, R (T), was measured using the standard four-probe (in a line, L_v_ = 1 cm, L_i_ = 0.8 cm) technique with the Resistivity model. Hall effect measurements were carried out with Ecopia HMS 3000 setup with a constant current of 0.3 mA in a 4-point configuration measuring an area of 0.5 cm^2^. The work function of Au and NbS_2_ film were determined by ultraviolet photoemission spectroscopy in Kratos Axis Ultra DLD multitechnique surface analysis system using He I (21.21 eV) photon lines from a discharge lamp with an error about 0.01 eV. The photocurrent density–voltage characteristics of the solar cells were measured using a 1000 W xenon solar simulator (Newport), and the light intensity was calibrated to 100 mW cm^−2^ by a NREL-calibrated Si cell (Oriel 91150). All devices were measured under the ambient air condition at room temperature (24 °C). The active area is 0.16 cm^2^ defined by a black mask. The IPCE spectrum was measured by a Newport QE system equipped with a 300 W xenon lamp.

## Electronic supplementary material

Supplementary Information
