# Supplementary material for: 2H-NbS2 film as a novel counter electrode for meso-structured perovskite solar cells
Source: Sci Rep. 2018 May 4;8:7033. doi: 10.1038/s41598-018-25449-x (PMC5935697; doi:10.1038/s41598-018-25449-x)
Supplement: Supplementary file 1 — Supplementary Information [file 41598_2018_25449_MOESM1_ESM.pdf]

Supplementary Information

$2H\text{-}NbS_2$ film as a novel counter electrode for meso-structured perovskite solar cells

Feng Shao,[1,2] Zhangliu Tian,[2] Peng Qin,[2,*] Kejun Bu,[2] Wei Zhao,[2] Li Xu,[4] Deliang Wang,[1,*] and Fuqiang Huang[2,3,*]

[1] Hefei National Laboratory for Physical Sciences at the Microscale, University of Science and Technology of China, Hefei 230026, China

[2] State Key Laboratory of High Performance Ceramics and Superfine Microstructure, Shanghai Institute of Ceramics, Chinese Academy of Sciences, Shanghai 200050, China.

[3] State Key Laboratory of Rare Earth Materials Chemistry and Applications, College of Chemistry and Molecular Engineering, Peking University, Beijing 100871, China

[4] Material Laboratory of State Grid Corporation of China, State Key laboratory of Advanced Transmission Technology, Global Energy Interconnection Research Institute, China

*** Corresponding Authors E-mail:**

Prof. Peng Qin: qinpeng@mail.sic.ac.cn

Prof. Deliang Wang: eedewang@ustc.edu.cn

Prof. Fuqiang Huang: huangfq@mail.sic.ac.cn

2H-NbS_2 Electronic Structure Calculation

DFT calculations were performed using the Vienna Ab Initio Simulation Package (VASP). The Perdew-Burke-Ernzehof (PBE) version of the generalized gradient approximation (GGA) was used to describe the exchange correlation functional, and the projector augmented wave (PAW) method was used in the present work. Here the cutoff energy of the plane wave was chosen at 480 eV. For the structure optimizations, $8 \times 8 \times 2$ k-points were used for the conventional cell. The convergence criteria are that the changes in total energies between two successive electronic steps are less than 10^{-5} eV, and all the Hellmann-Feynman force acting on each atom is less than 0.01 eV/Å.

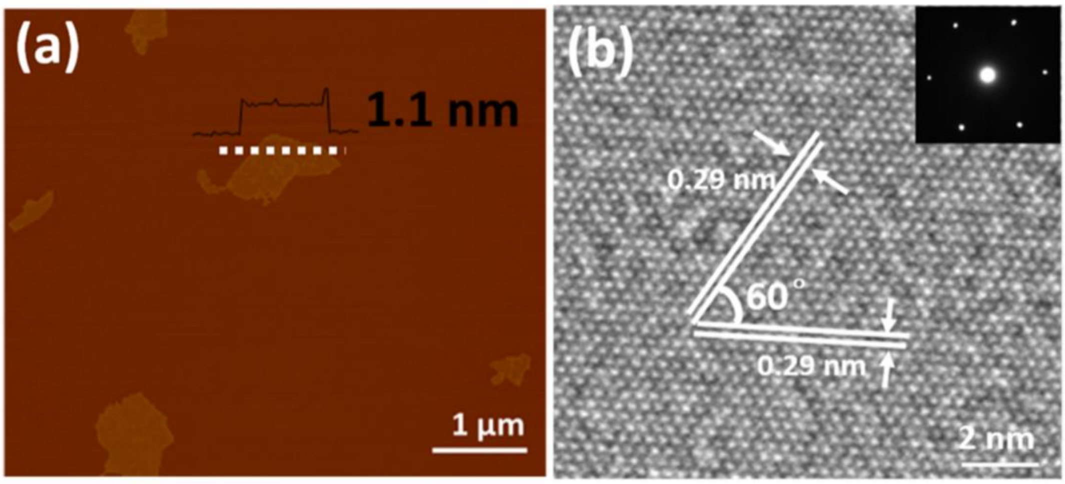

Figure. S1 The atomic force microscopy (AFM) image and (b) high resolution transmission electron microscopy (HRTEM) image of the NbS_2 nanosheets obtained from Li_xNbS_2 powder (inset is the selected area electron diffraction (SEAD) pattern).

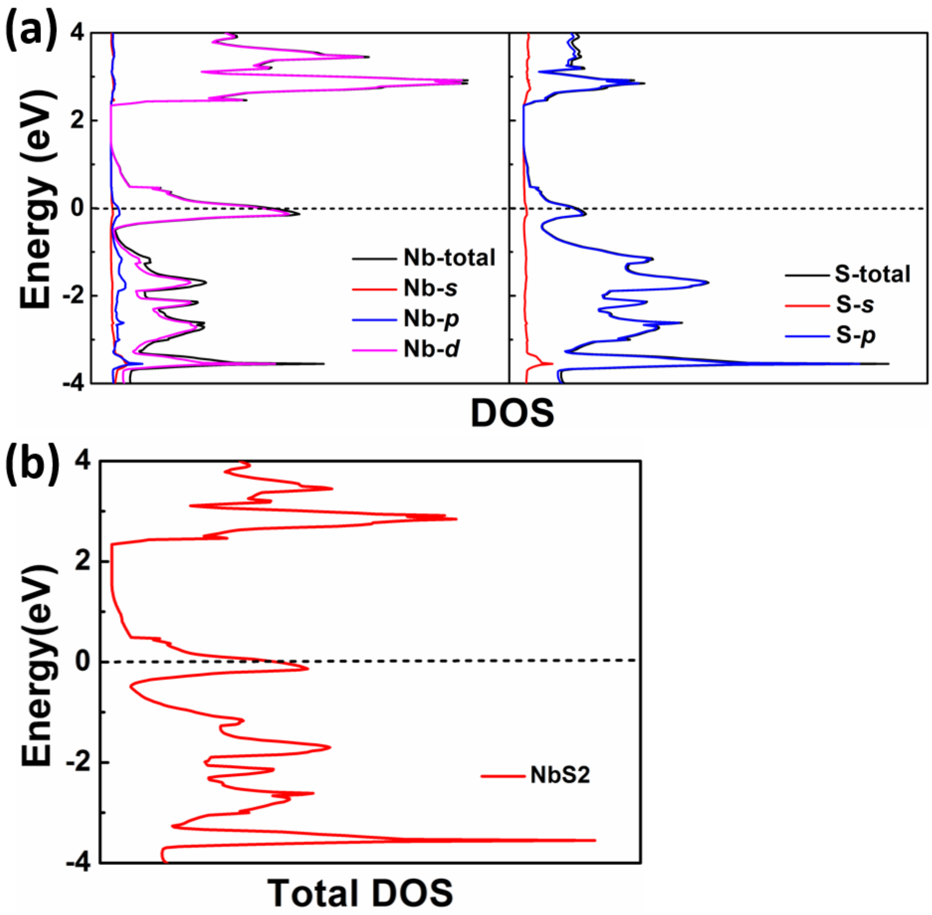

Figure. S2 (a) Partial DOS and (b) total DOS of the restacked 2H-NbS_2 film.

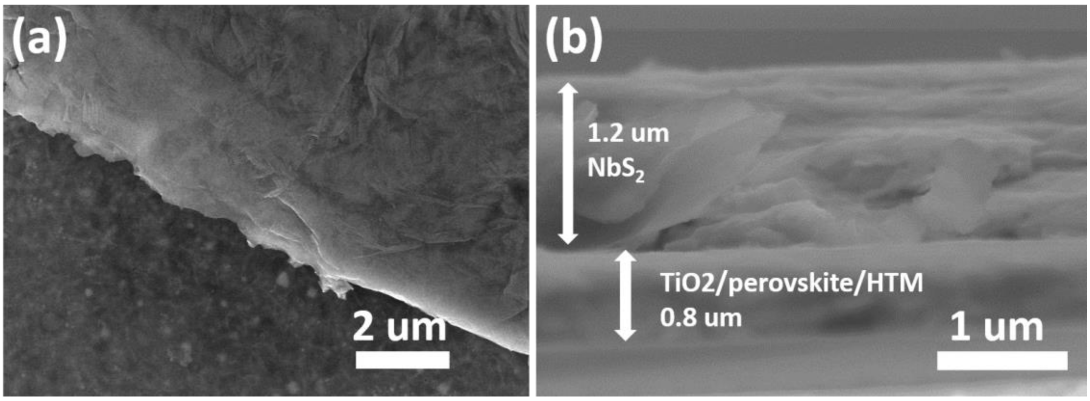

Figure. S3 The edge morphology of the NbS_2 counter electrode plastered on the spiro-OMeTAD surface after a 280 MPa isostatic pressing treatment. (b) Cross-sectional FESEM image of the device with NbS_2 film as counter electrode.

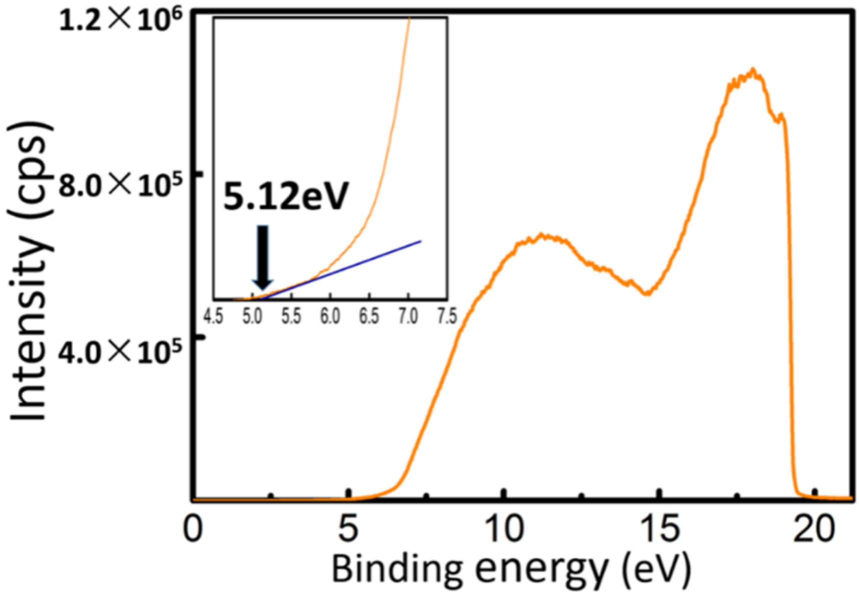

Figure. S4 UPS spectrum of the thermal evaporated Au film with a thickness of 100nm. The inset is the magnification of local detail.

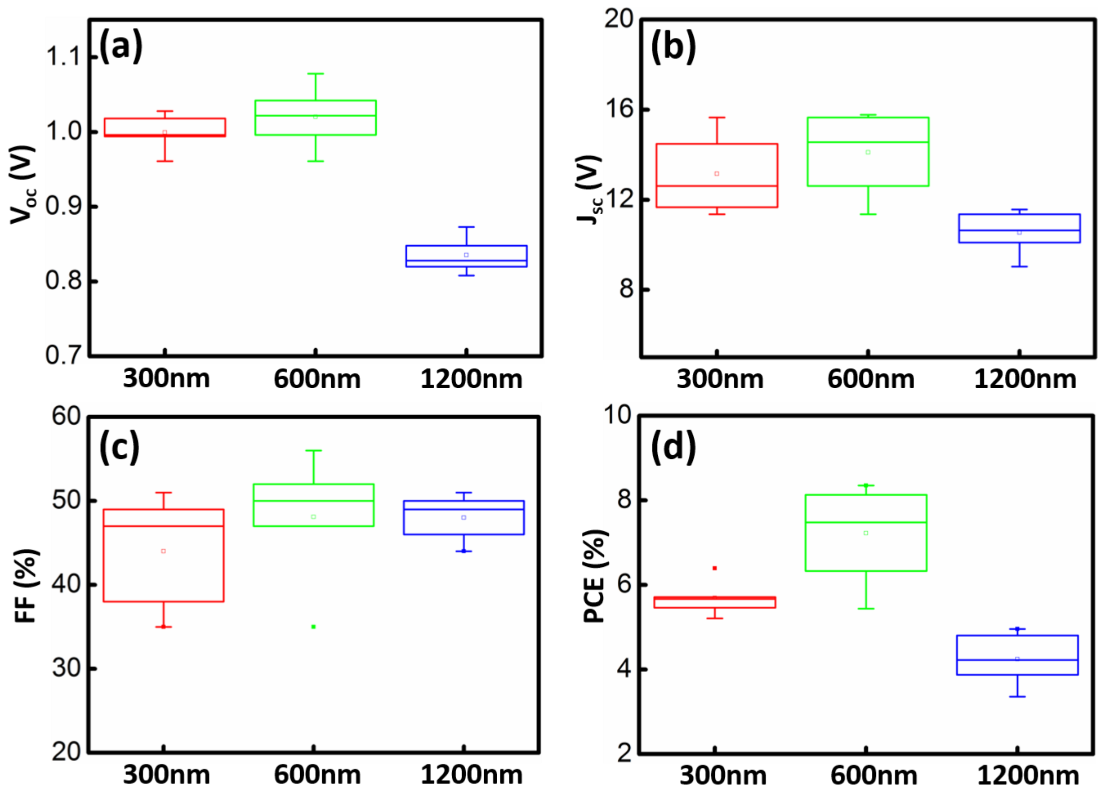

Figure. S5 Photovoltaic parameters (V_{oc}, J_{sc}, FF, and PCE) distribution shown as box plots for the devices based on NbS_2 electrodes with different thicknesses. Each box represents the distribution of 10 devices prepared under the same condition.

Table. S1 The photovoltaic parameters of the perovskite solar cells based on NbS_2 electrodes with different thicknesses. Each value of thickness of NbS_2 electrode contain 10 devices prepared under the same conditions.

Sample	V_{oc} (mV)	J_{sc} (mA cm^{-2})	*FF* (%)	η (%)
300 nm	999	13.15	44	5.69
600 nm	1020	14.11	48	7.22
1200 nm	835	10.54	48	4.24

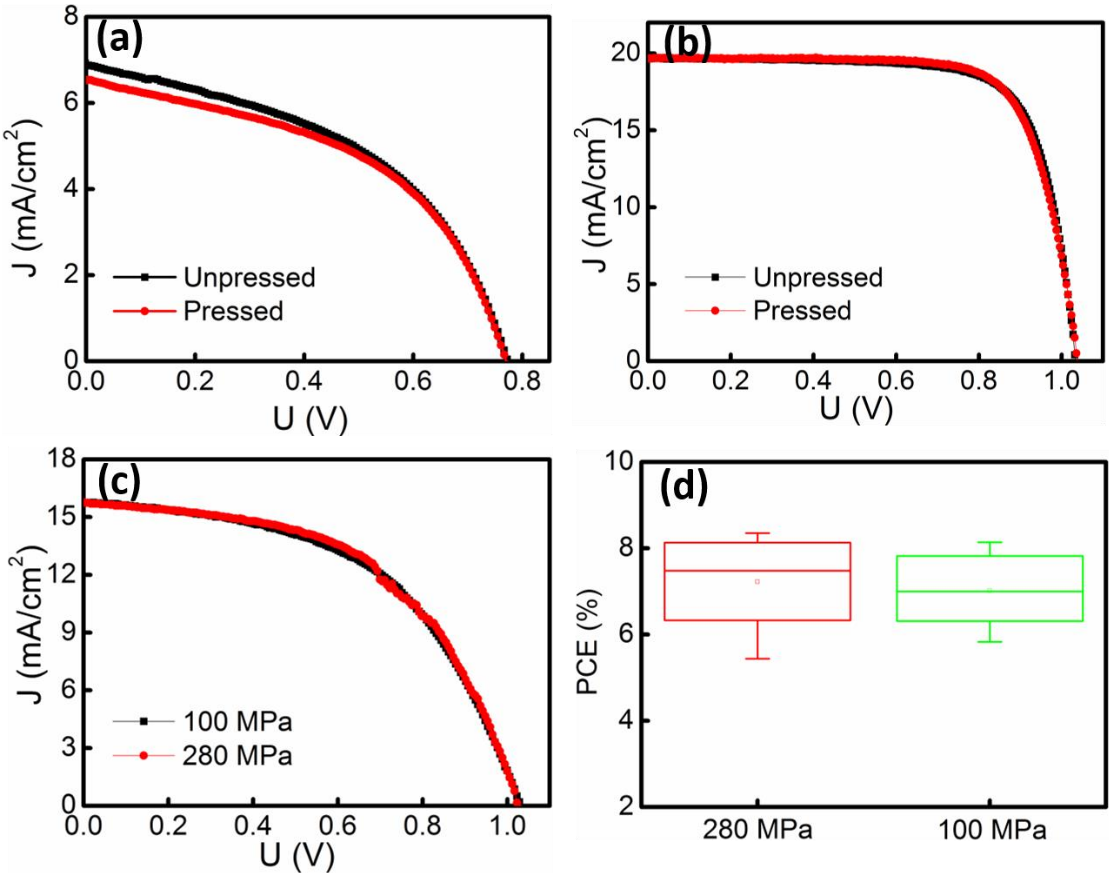

Figure. S6 Current-voltage (*J*-*V*) curves of (a) TiO_2/perovskite/Au and (b) TiO_2/perovskite/spiro-OMeTAD/Au devices before (black) and after (red) 280 MPa pressing treatment under AM 1.5 full sun illumination. (c) Current-voltage (*J*-*V*) curves of the solar cells based on NbS_2 electrode by the CIP method with the pressure of 100 MPa (black), and once again at 280 MPa (red). (d) PCE distribution shown as box plots for the NbS_2 based devices prepared at different pressure. Each box represents the distribution of 5 devices prepared under the same condition.

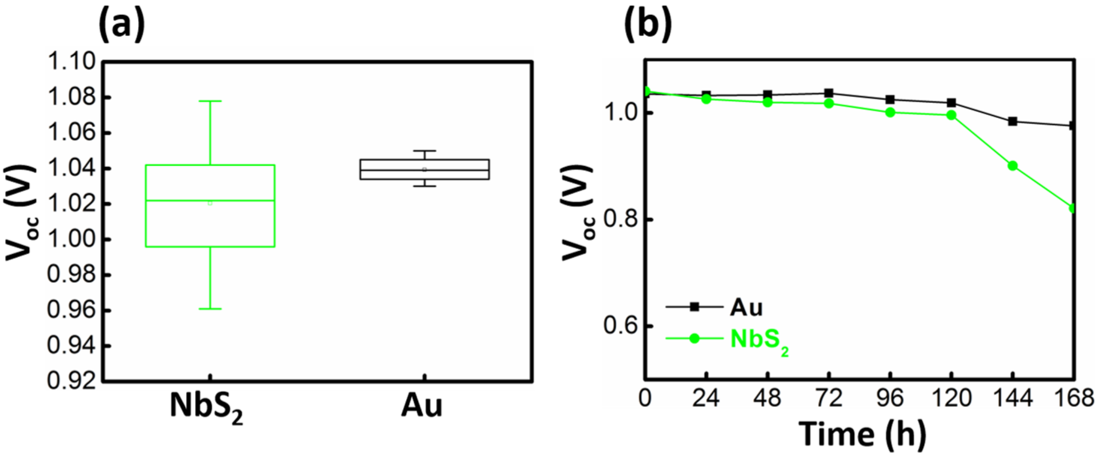

Figure. S7 (a) Statistics of V_{oc}. Each box represents the distribution of 10 devices prepared under the same conditions. (b) Degradation of V_{oc} of the unencapsulated two best-performed devices based on NbS_2 and Au counter electrode under the ambient condition at room temperature (24℃).
